# A eukaryotic-like 3′ untranslated region in *Salmonella enterica hilD* mRNA

**DOI:** 10.1093/nar/gku222

**Published:** 2014-03-20

**Authors:** Javier López-Garrido, Elena Puerta-Fernández, Josep Casadesús

**Affiliations:** Departamento de Genética, Universidad de Sevilla, Facultad de Biología, Apartado 1095, 41080 Sevilla, Spain

## Abstract

Long 3′ untranslated regions (3′UTRs) are common in eukaryotic mRNAs. In contrast, long 3′UTRs are rare in bacteria, and have not been characterized in detail. We describe a 3′UTR of 310 nucleotides in *hilD* mRNA, a transcript that encodes a transcriptional activator of *Salmonella enterica* pathogenicity island 1 (SPI-1). Deletion of the *hilD* 3′UTR increases the *hilD* mRNA level, suggesting that the *hilD* 3′UTR may play a role in *hilD* mRNA turnover. Cloning of the *hilD* 3′UTR downstream of the green fluorescent protein (*gfp*) gene decreases green fluorescent protein (GFP) activity in both *Escherichia coli* and *S. enterica*, indicating that the *hilD* 3′UTR can act as an independent module. *S. enterica* mutants lacking either ribonuclease E or polynucleotide phosphorylase contain similar amounts of *hilD* and *hilD* Δ3′UTR mRNAs, suggesting that the *hilD* 3′UTR is a target for *hilD* mRNA degradation by the degradosome. The *hilD* 3′UTR is also necessary for modulation of *hilD* and SPI-1 expression by the RNA chaperone Hfq. Overexpression of SPI-1 in the absence of the *hilD* 3′UTR retards *Salmonella* growth and causes uncontrolled invasion of epithelial cells. Based on these observations, we propose that the *S. enterica hilD* 3′UTR is a *cis*-acting element that contributes to cellular homeostasis by promoting *hilD* mRNA turnover.

## INTRODUCTION

Posttranscriptional regulation of gene expression plays essential roles in cellular physiology in all domains of life. During the last few decades, special attention has been paid to mRNA regulation, and a variety of regulatory mechanisms have been described in both prokaryotes and eukaryotes. A difference in posttranscriptional regulation between eukaryotes and prokaryotes is the region of the mRNA molecule involved: while in eukaryotes the interaction of proteins or RNA molecules occurs with sequences located either in the 5′ untranslated region (5′UTR) or in the 3′ untranslated region (3′UTR) (1), posttranscriptional regulation in prokaryotes is most commonly exerted by targeting either the 5′UTR or the promoter-proximal region of the coding sequence (2–4).

*Salmonella enterica* is a Gram-negative bacterium that has played a historic role in bacterial genetics and in the study of host–pathogen interactions. More recently, *Salmonella* has also become a model organism for the study of RNA-mediated regulation (5). A relevant trait of *Salmonella* virulence is the ability of the pathogen to enter epithelial cells in the animal intestine. This process, known as invasion, requires translocation of bacterial proteins from the bacterial cytoplasm to the epithelial cell cytoplasm through a type 3 secretion system (TTSS) (6,7). The translocated proteins or ‘effectors’ interact with specific targets inside the epithelial cell, triggering a cascade of molecular events that permit *Salmonella* invasion (6).

Invasion effectors and components of the cognate TTSS are encoded in a 40 kb region of the *Salmonella* chromosome known as *Salmonella* pathogenicity island 1 (SPI-1) (7–9). Expression of SPI-1 genes is coordinately regulated by environmental signals and cellular factors through a regulatory network of SPI-1-encoded transcriptional activators, and one of the main transcription factors is HilD (8–10). A number of regulators seem to control *hilD* expression at the posttranscriptional or the posttranslational level, rather than at the level of transcription initiation (10), and the underlying mechanisms are known in certain cases only. For instance, HilE, a negative SPI-1 regulator (11), physically interacts with HilD (12), likely interfering with its function. Another regulator, FliZ, may activate SPI-1 expression by controlling HilD activity (13). Regulation of *hilD* expression at the mRNA level has also been proposed: overproduction of the RNA binding protein CsrA represses SPI-1 expression (14,15), and CsrA has been shown to bind a *hilD* mRNA region that overlaps with the ribosome binding sequence, likely preventing translation and accelerating mRNA decay (15). DNA adenine (Dam) methylation contributes to sustain high levels of SPI-1 expression by decreasing *hilD* mRNA turnover but the mechanism remains to be identified (16).

Here we show that *hilD* mRNA possesses a long 3′UTR of 310 nucleotides, and provide evidence that the *hilD* 3′UTR is a target for the RNA degradosome and for the RNA chaperone Hfq. Lack of the *hilD* 3′UTR permits SPI-1 expression under conditions that are not favorable for invasion and retards *Salmonella* growth, suggesting that the *hilD* 3′UTR may have physiological significance.

## MATERIALS AND METHODS

### Bacterial strains, bacteriophages and standard strain construction

All the *S. enterica* strains listed in Supplementary Table S1 belong to serovar Typhimurium, and derive from the mouse-virulent strain ATCC 14028. For simplicity, *S. enterica* serovar Typhimurium is usually abbreviated as *S. enterica.* Two *Escherichia coli* strains were used: DH5α [F^−^ Φ80*lacZΔM15 Δ(lacZYA-argF*) *U169 recA1 endA1 hsdR17 (rK^−^, mK^+^) phoA supE44 λ^−^ thi-1 gyrA96 relA1*] and CC118 *λ pir* [*phoA20 thi-1 rspE rpoB argE(Am) recA1 (λ pir)*]. Targeted gene disruption was achieved using pKD4 or pKD13 (17). Antibiotic resistance cassettes introduced during strain construction were excised by recombination with plasmid pCP20 (17). The oligonucleotides used for disruption (labeled ‘UP’ and ‘DO’) are listed in Supplementary Table S2, together with the oligonucleotides (labeled ‘E’) used for allele verification by the polymerase chain reaction. For construction of *lac* fusions in the *Salmonella* chromosome, FLP recombinase recognition target (FRT) sites generated by excision of Km^r^ cassettes (17) were used to integrate either pCE37 or pCE40 (18). Unless indicated otherwise, all *lac* fusions used in this study are translational. Addition of a 3xFLAG epitope tag to protein-coding DNA sequences was carried out using plasmid pSUB11 (Km^r^, 3xFLAG) as template (19). Transductional crosses using phage P22 HT 105/1 *int201* [(20) and Roberts,G., unpublished data] were used for strain construction operations involving chromosomal markers. The transduction protocol was described elsewhere (21). To obtain phage-free isolates, transductants were purified by streaking on green plates. Phage sensitivity was tested by cross-streaking with the clear-plaque mutant P22 H5.

### Media and growth conditions

Luria-Bertani (LB) broth was used as standard liquid medium. For standard conditions (SPI-1 induction) (22), cultures were grown in LB at 37°C with shaking, and samples were taken when the cultures had reached stationary phase (O.D. = 2–2.5). To measure SPI-1 expression under non-invasive conditions, saturated cultures were diluted 1:50 in two different conditions: (i) LB and incubation at 37°C with shaking (200 rpm) until O.D._600_ = 0.6; (ii) LB without salt and incubation at 37°C with shaking (200 rpm) until O.D._600_ = 2–2.5. When required, kanamycin sulfate (50 μg/ml) or chloramphenicol (20 μg/ml) were added to the culture medium. Green plates were prepared according to Chan *et al.* (23), except that methyl blue (Sigma Chemical Co., St Louis, MO) substituted for aniline blue.

### Construction of relevant strains

For construction of the *hilD* Δ3′UTR allele, the Km^r^ gene of pKD13 was amplified with primers JVO5462 and JVO5463 (Supplementary Table S2) and inserted in the *Salmonella* chromosome by λ Red recombination, deleting a 230 nt fragment of the *hilD* 3′UTR starting at the first nucleotide after the *hilD* stop codon. The Km^r^ gene was removed by recombination of flanking FRT sequences mediated by pCP20-encoded FLP recombinase, leaving a scar of 82 nt. As a result, a shorter 3′UTR of 162 nt was produced without eliminating the native Rho-independent transcriptional terminator. The same strategy was used to construct *hilD* 3′UTR deletion alleles, using different forward primers (Supplementary Table S2) and keeping JVO5463 as the reverse oligo for amplification. The *hilD* 3′UTR_50end_ allele was constructed using primer pairs JVO5462 and hilD50endDO.

The *hilD* Δ3′UTRsl (scarless) allele was constructed by overlapping polymerase chain reactions using oligonucleotides EPF097, EPF100, EPF103 and EPF104 (Supplementary Table S2). The resulting amplicon contained the *hilD* coding sequence followed by the 3′UTR transcriptional terminator (without the 3′UTR) and approximately 500 bp of nearby DNA, flanked by SacI and XbaI restriction sites. The polymerase chain reaction (PCR) product was cloned into the SacI–XbaI sites of the suicide plasmid pDMS197, and propagated in *E. coli* CC118 lambda *pir*. The pDMS197 derivative was transformed into *E. coli* S17-1 lambda *pir*, and several transformants were used as donors in matings with *S. enterica* 14028 harboring a Km^R^ cassette in place of the *hilD* 3′UTR (strain SV6189). Tc^R^ transconjugants were selected on E plates supplemented with tetracycline. Several Tc^R^ transconjugants were grown in nutrient broth (without NaCl) containing 5% sucrose. Individual tetracycline-sensitive segregants were then screened for kanamycin sensitivity and examined for the incorporation of the mutant *hilD* allele (*hilD* Δ3′UTRsl) by PCR amplification with oligonucleotides EPF097 and EPF100 and sequencing of the PCR product.

Expression of *hilD* from a heterologous promoter was achieved by replacing its native promoter with the P_LtetO_ promoter (24). A fragment containing the *cat* gene and P_L*tetO*_ was amplified by PCR using pXG1 as template (25). The primers were labeled P_LtetO_-*hilD* UP and P_LtetO_-*hilD* DO (Supplementary Table S2). The PCR product was treated with DpnI to remove template traces. The construction was then inserted in the *S. enterica* chromosome by λ Red recombination (17) and Cm^r^ colonies were selected. Insertion of the construction was verified by PCR, using a pair of primers specific for the *cat* gene and the target gene (Supplementary Table S2).

Expression of *gfp* alleles was achieved by electroporation of pXG1-derived plasmids into *S. enterica* ATCC 14028. To generate the *gfp* allele carrying a *hilD* 3′UTR, a PCR product containing the *hilD* 3′UTR coding sequence downstream of *gfp* was generated by overlapping PCRs. Two PCR products were obtained using primer pairs EPF043–EPF044 (Supplementary Table S2) and pXG1 as template, and primers EPF045 and EPF046 (Supplementary Table S2) and genomic DNA from *S. enterica* ATCC 14028 as template. Purified PCR products were subsequently used as templates for amplification with primers EPF043 and EPF046. The resulting product was cloned into *Nhe*-I digested pXG1 vector, rendering plasmid pIZ1988. To generate the *gfp* allele with a 3′UTR deletion (Δ3′UTR) the same procedure was used. The primer pairs used were EPF043 and EPF047 (Supplementary Table S2), with pXG1 as template, and primers EPF048 and EPF046 (Supplementary Table S2) with SV6190 genomic DNA as template. The final PCR product was cloned into pXG1 to obtain pIZ1989. Plasmids pIZ1988 and pIZ1999 were constructed using *E. coli* DH5α as host. Transfer to *S. enterica* was achieved by electrotransformation of ATCC 14028, selecting Cm^r^ colonies. Introduction of pIZ1988 and pIZ1999 generated strains SV7440 and SV7441, respectively.

### RNA extraction procedures

A 2 ml aliquot from a culture of the appropriate strain grown to stationary phase (O.D._600_ ∼ 2) was centrifuged at 16 000 *g*, 4°C, for 5 min. The pellet was resuspended in 100 μl of a solution of lysozyme (3 mg/ml, Sigma Chemical Co.). Cell lysis was facilitated by three consecutive freeze-thaw cycles. After lysis, RNA was extracted using 1 ml of Trizol reagent (Invitrogen Co., Carlsbad, CA), according to manufacturer's instructions. Finally, total RNA was resuspended in 30 μl of RNase-free water for subsequent uses. The quality of the preparation and the RNA concentration were determined using a ND-1000 spectrophotometer (NanoDrop Technologies, Wilmington, DE).

### 3′RACE

Rapid amplification of cDNA 3' ends (3′RACE) was performed as described by Argaman *et al.* (26). The *hilD-*specific primer used for PCR was JVO5527. Specific PCR products were cloned using the TOPO TA cloning (Invitrogen Co., Carlsbad, CA), and four independent clones of each product were sequenced using external primers.

### Northern blotting

Ten micrograms of RNA was loaded per well and electrophoresed in denaturing 1% agarose formaldehyde–3-(N-morpholino)propanesulfonic acid (MOPS) gels. Transfer and fixation to Hybond-N^+^ membranes (GE Healthcare, Little Chalfont, UK) were performed by vacuum using 0.05 M NaOH. Filters were then hybridized using an internally labeled [(α-^32^P)UTP] riboprobe specific for the first (5′) 300 nucleotides of the *hilD* coding sequence. Hybridization was carried out at 65°C. As a control of RNA loading and transfer efficiency, the filters were hybridized with a riboprobe for the RNase P mRNA gene (*rnpB*). Images of radioactive filters were obtained with a FLA-5100 imaging system (Fujifilm, Tokyo, Japan), and quantification was performed using the MultiGauge software (Fujifilm).

### β-Galactosidase assays

Levels of β-galactosidase activity were assayed using the CHCl_3_–sodium dodecyl sulfate (SDS) permeabilization procedure (27). Unless otherwise indicated, the results shown are averages and standard deviations from three independent experiments.

### Protein extracts and western blot analysis

Total protein extracts were prepared from bacterial cultures grown at 37°C in LB until stationary phase (final O.D._600_ ∼ 2.5). Approximately 3.5 × 10^8^ bacterial cells were collected by centrifugation (16 000 *g*, 2 min, 4°C) and suspended in 100 μl of Laemmli sample buffer [1.3% SDS, 10% (v/v) glycerol, 50 mM Tris–HCl, 1.8% β-mercaptoethanol, 0.02% bromophenol blue, pH 6.8]. Proteins were resolved by Tris–Tricine–polyacrylamide gel electrophoresis (PAGE) using 12% gels. Conditions for protein transfer have been described elsewhere (28). Optimal dilutions of primary antibodies were as follows: anti-Flag M2 monoclonal antibody (1:5000, Sigma Chemical Co.), and anti-GroEL polyclonal antibody (1:20 000, Sigma Chemical Co.). Goat anti-mouse horseradish peroxidase-conjugated antibody (1:5000, BioRad, Hercules, CA) or goat anti-rabbit horseradish peroxidase conjugated antibody (1:20 000, Santa Cruz Biotechnology, Heidelberg, Germany) were used as secondary antibodies. Proteins recognized by the antibodies were visualized by chemoluminescence using the luciferin–luminol reagents (Pierce Reagents), and a LAS3000 mini imaging system (Fujifilm). For quantification, the intensity of the bands was determined using MultiGauge software (Fujifilm).

### Invasion assays

HeLa cells (ATCC CCL2) were cultured in tissue culture medium (Dulbecco's modified essential medium supplemented with 10% fetal calf serum and 2 mM l-glutamine). For routine cultivation, 60 μg/ml penicillin and 100 μg/ml streptomycin were added to the culture medium. The day before infection, ∼1.5 × 10^5^ HeLa cells were seeded, using 24-well plates (Costar, Corning, New York, NY). Each well contained 1 ml of tissue culture medium without antibiotics. Cells were grown at 37°C, 5% CO_2_ to obtain 80% confluency. One hour before infection, the culture medium was removed and replaced by 0.5 ml fresh tissue culture medium without antibiotics. Bacteria were grown in LB to an O.D. ≈ 0.4 and added to reach a multiplicity of infection (MOI) of 50:1 bacteria/HeLa cell. HeLa cells were infected for 30 min, washed three times with PBS, incubated in fresh tissue culture medium containing 100 μg/ml gentamicin for 1.5 h, and washed three times with PBS. Numbers of viable intracellular bacteria were obtained by lysing infected cells with 1% Triton X-100 (prepared in PBS) and subsequent plating. Invasion rates were determined as the ratio between viable intracellular bacteria and viable bacteria added to infect the HeLa cells. Each assay was run in triplicate, and data shown are the relative invasion rates and standard deviations of five independent experiments. The Student's *t* test was used to determine the statistical significance of the differences observed.

### Flow cytometry

GFP expression was monitored by flow cytometry of live cells. Bacteria were grown overnight at 37°C in LB with shaking, diluted into fresh medium (1:100), and incubated at 37°C to reach and O.D._600_ ∼ 2. For each sample, the GFP fluorescence of at least 50 000 cells was measured using a Cytomics FC500-MPl flow cytometer (Beckman Coulter, Brea, CA).

## RESULTS

### *hilD* mRNA has a long 3′UTR

**Figure 1.**
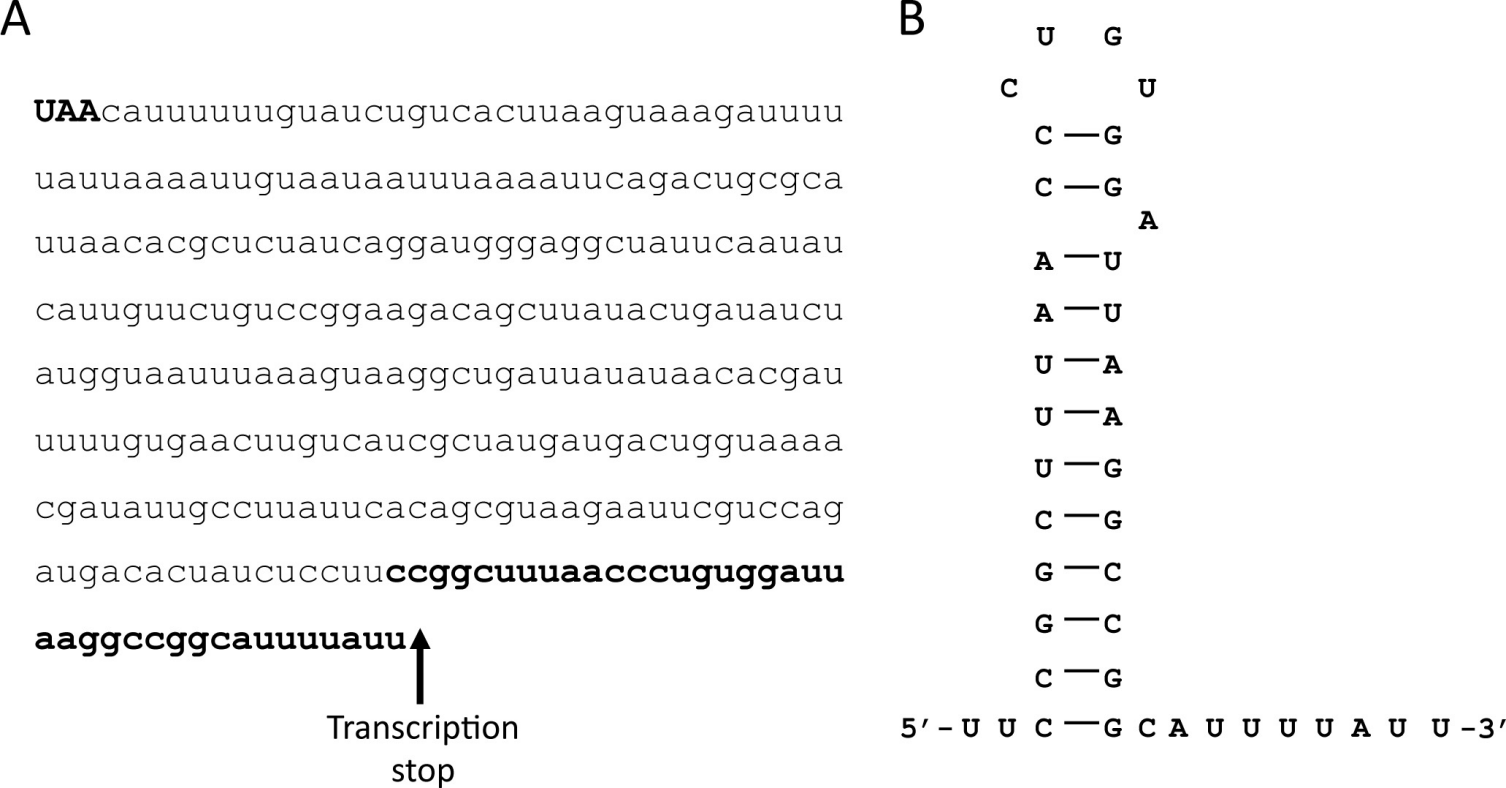
(**A**) Nucleotide sequence of the *hilD* 3′UTR. The first three nucleotides (in bold capital letters) correspond to the *hilD* stop codon. Nucleotides in bold at the 3′ end of the sequence constitute the Rho-independent transcriptional terminator depicted in panel (B). The arrow indicates the *hilD* transcription termination point determined by 3′RACE. (**B**) Stem-loop structure of the Rho-independent transcriptional terminator predicted by RNAfold software (bold nucleotides in panel (A)).

The *hilD* transcription start point was identified previously (29), defining a 5′UTR of 35 nucleotides. In turn, *in silico* annotation of the *S. enterica* genome identified a putative long intergenic region between *hilD* and the neighbor gene *hilA* (http://www.xbase.ac.uk/colibase/). We determined the *hilD* transcription stop point by 3′RACE and mapped the *hilD* transcriptional stop around 300 nucleotides downstream the *hilD* stop codon, immediately after a U-rich region (Figure 1A; Supplementary Figure S1). U-rich tracts are typically found at the 3′ end of Rho-independent transcriptional terminators (30,31). Furthermore, sequence analysis with the RNAfold software (32–34) predicts the existence of a secondary structure similar to a Rho-independent transcriptional terminator which includes the U-rich region defined by 3′RACE in the *hilD* transcript (Figure 1B). Altogether, these data provide evidence that *hilD* mRNA may be 1275 nucleotides long, including a 5′UTR of 35 nucleotides, a coding sequence of 930 nucleotides, and an unusually long 3′UTR of 310 nucleotides. Northern blot analysis in a 4.5% polyacrylamide gel with a riboprobe specific for *hilD* identified an RNA molecule of ∼1.3 kb, consistent with the estimated size of *hilD* mRNA (Supplementary Figure S2). *In silico* analysis of the 3′UTR (35) did not reveal the existence of any potential coding sequence: the longest open reading frame (ORF), 57 nucleotides long, is not preceded by a putative ribosome binding site (data not shown). Nevertheless, the region is conserved among *Salmonella* serovars (Supplementary Figure S3), which might indicate a functional role for this non-coding RNA region.

### Deletion of the *hilD* 3′UTR increases the level of *hilD* mRNA

**Figure 2.**
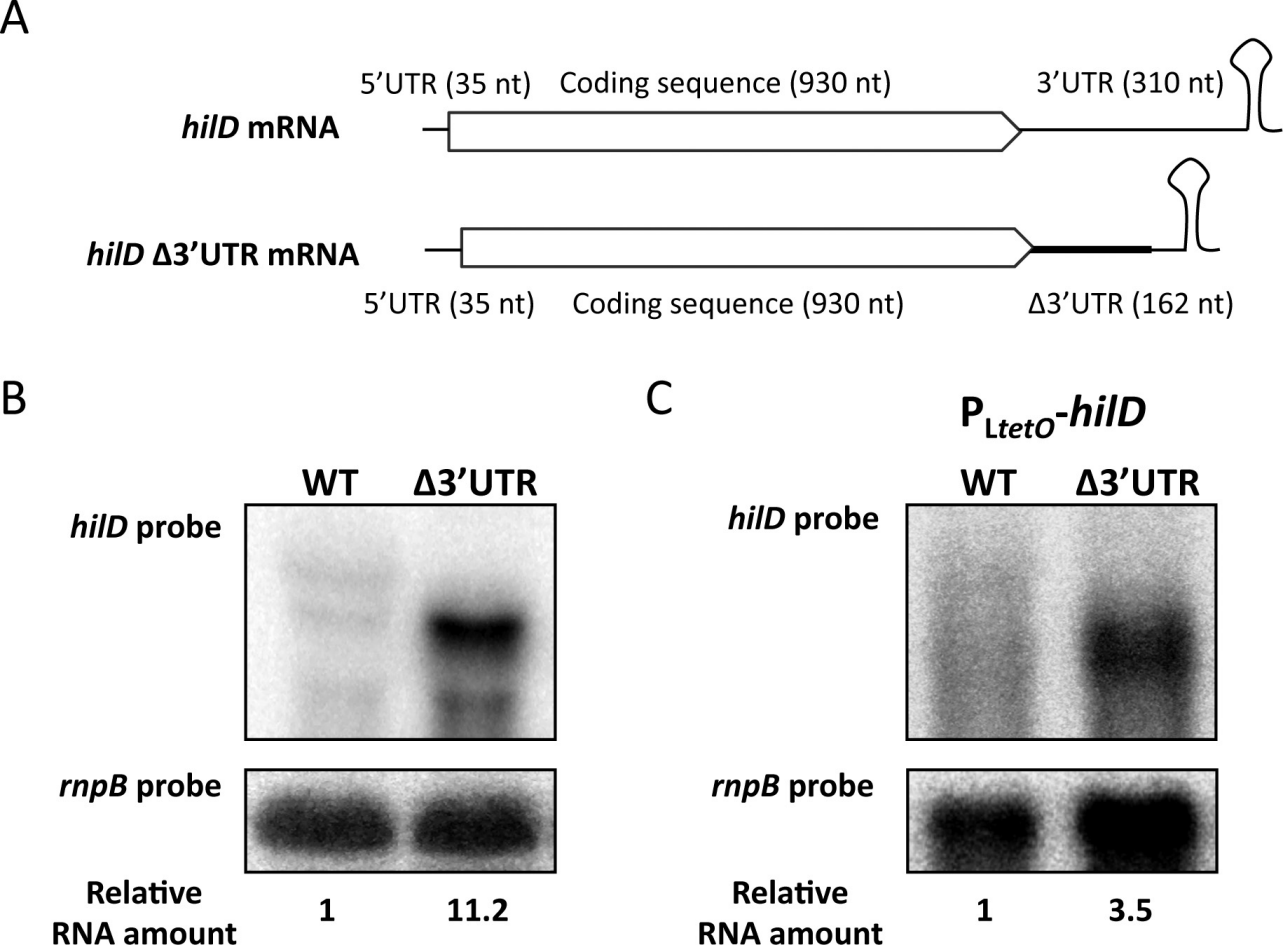
(**A**) Diagrams of *hilD* mRNA and *hilD* Δ3′UTR mRNA. The thick line in the 3′ region of *hilD* Δ3′UTR mRNA represents the 82-nt pKD13 scar. The Rho-independent transcriptional terminator is left intact in the constructs. (**B** and **C**) Northern blots of *hilD* and *hilD* Δ3′UTR mRNAs when *hilD* is transcribed from its own promoter (B), and when transcription is driven by the P_L*tetO*_ promoter (C). Three discrete bands are detected with a riboprobe when *hilD* is transcribed from its native promoter. *rnpB* mRNA has been used as loading control. For quantification, the ratio *hilD/rnpB* was normalized to 1 in *hilD* mRNA carrying a native 3′UTR.

Long 3′UTRs have been poorly studied in prokaryotes, while eukaryotic 3′UTRs often play physiological roles (36). We thus considered the possibility that the *hilD* 3′UTR might play a role in *hilD* expression. To test this hypothesis, we constructed a strain carrying a modified *hilD* 3′UTR (*hilD* Δ3′UTR). In this strain, a 230 nt fragment of the *hilD* 3′UTR, starting at the first nucleotide after the *hilD* stop codon, was deleted leaving the Rho-independent transcriptional terminator intact. The deleted fragment was replaced with the pKD13 scar (82 nucleotides) (17), resulting in a shorter 3′UTR with different sequence but conserving the native transcriptional terminator (Figure 2A). To test whether the presence of the *hilD* 3′UTR altered the *hilD* mRNA level, we isolated total RNA from the wild type and the *hilD* Δ3′UTR strain and monitored *hilD* expression by northern blotting using a ^32^P-labeled riboprobe specific for the upstream (5′) 300 nucleotides of the *hilD* coding sequence. As shown in Figure 2B, the *hilD* Δ3′UTR mRNA is shorter than the *hilD* native mRNA. More importantly, the level of *hilD* mRNA was found to be ∼11-fold higher in the absence of the 3′UTR than in the wild-type strain, suggesting that the presence of the 3′UTR somehow reduces the *hilD* mRNA level. To exclude the possibility that the *hilD* mRNA increase detected in the *hilD* Δ3′UTR strain could be caused by a stabilizing effect of the scar sequence, we constructed a strain in which the 3′UTR was cleanly deleted. Higher levels of *hilD* mRNA were detected in the scarless strain (Supplementary Figure S4). The specificity of the *hilD* probe was confirmed using a strain carrying a SPI-1 deletion, and no signal was detected by northern blot (Supplementary Figure S4).

To test whether the *hilD* 3′UTR is involved in *hilD* expression, we compared the levels of *hilD* mRNA in isogenic *hilD* 3′UTR^+^ and *hilD* Δ3′UTR strains in which *hilD* was transcribed from a heterologous promoter, P_L*tetO*_ (24). Absence of the native 3′UTR increased the *hilD* mRNA level 3.5-fold (Figure 2C). Because this observation was made in strains that expressed *hilD* from a heterologous promoter, we tentatively concluded that the 3′UTR influences *hilD* expression at the posttranscriptional level. Note that *hilD* expression upon deletion of the 3′UTR is higher when *hilD* is transcribed from its own promoter than when transcription is driven by P_L*tetO*_ (Figure 2B and C). This observation is consistent with the occurrence of autogenous control of *hilD* transcription (37), which can be expected to create a positive feedback loop that amplifies the regulatory effect (38).

### The *hilD* 3′UTR reduces expression of a heterologous gene

**Figure 3.**
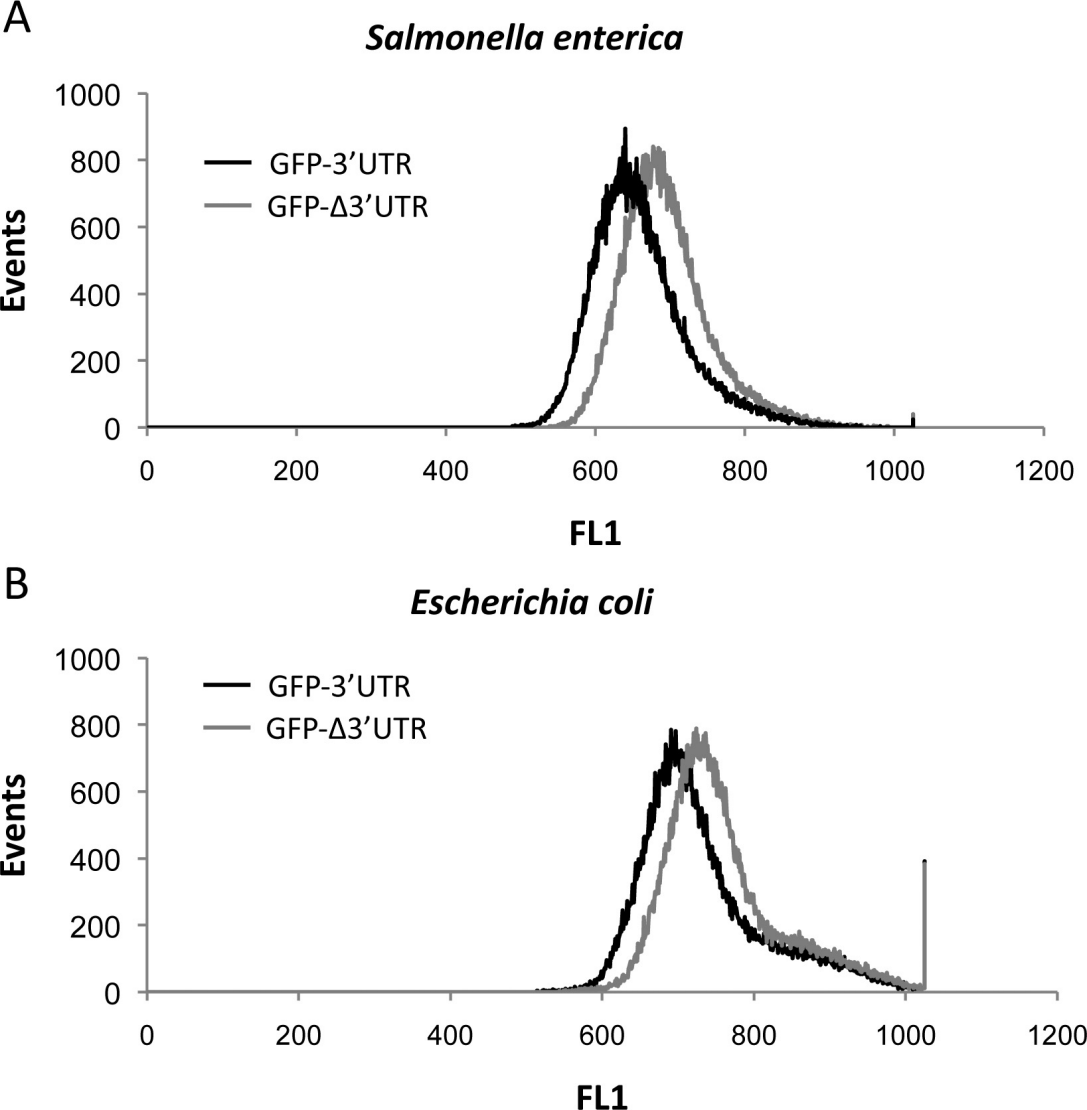
Histogram plots showing the fluorescence of *Salmonella enterica* (**A**) or *Escherichia coli* cells (**B**) carrying the *gfp* gene with either the *hilD* 3′UTR (GFP-3′UTR, solid line) or the *hilD* Δ3′UTR (GFP-Δ3′UTR, dashed line) downstream of the *gfp* stop codon. Over 50 000 particles were analyzed for each sample using a Cytomics FC500-MPl flow cytometer. Three independent experiments were performed, and the histogram plots depicted are representative of the results obtained. Analysis of data was performed using the CXP software.

To further confirm that the *hilD* 3′UTR has functional capacity, we analyzed the effect of the 3′UTR on the expression of a heterologous gene. A synthetic mRNA was generated by cloning the *hilD* 3′UTR downstream of the green fluorescent protein (*gfp*) gene on plasmid pXG1 (25), yielding plasmid pIZ1998. The synthetic mRNA was expressed constitutively from the P_L*tetO*_ promoter (24). As a control, a plasmid containing the *hilD* Δ3′UTR downstream of *gfp* (pIZ1989) was also constructed. Both plasmids were introduced in *S. enterica* and *E. coli*, and GFP levels were monitored in single bacterial cells by flow cytometry. A representative experiment is shown in Figure 3. *Salmonella enterica* cells presented a mean fluorescence of 58.4 for cells expressing GFP-3′UTR and 85.3 for cells expressing GFP-Δ3′UTR. In turn, *E. coli* cells showed a mean fluorescence of 96.9 for cells containing GFP-3′UTR and 132.0 for cells lacking the 3′UTR (GFP-Δ3′UTR). Thus, the presence of the *hilD* 3′UTR downstream of *gfp* resulted in lower fluorescence intensity (indicating lower GFP production) in both *S. enterica* and *E. coli*. This ability of the *hilD* 3′UTR to reduce expression of a heterologous gene supports the view that the 3′UTR can act as an independent module.

### Deletion of the *hilD* 3′UTR increases SPI-1 expression

**Figure 4.**
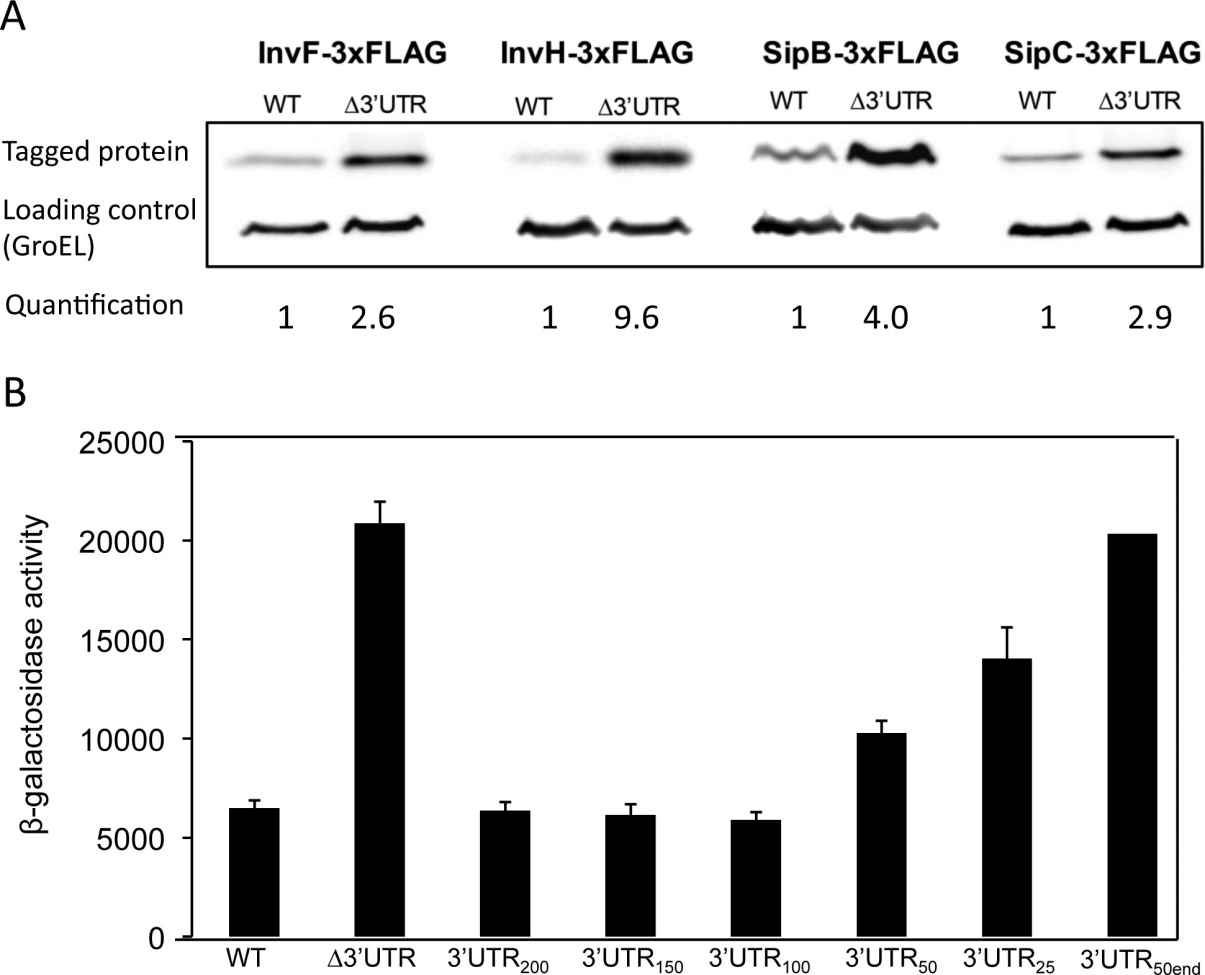
(**A**) Western blots of InvF-3xFLAG, InvH-3xFLAG, SipB-3xFLAG and SipC-3xFLAG in protein extracts from *hilD* 3′UTR^+^ and *hilD* 3′UTR^−^ hosts. GroEL was used as loading control. For quantification, the ratio 3xFLAG-tagged protein/GroEL was relativized to 1 in the *hilD* 3′UTR^+^ background. (**B**) β-Galactosidase activity of a *sipC::lac* fusion in *Salmonella enterica* strains containing a native *hilD* 3′UTR (wt) or alleles with deletions of the *hilD* 3′UTR (Δ3′UTR, 3′UTR_200_, 3′UTR_150_, 3′UTR_100_, 3′UTR_50_, 3′UTR_25_ and 3′UTR_50end_).

Because *hilD* mRNA is more abundant in the absence of its native 3′UTR and HilD is a positive regulator of SPI-1, we wondered whether increased level of *hilD* mRNA resulted in increased SPI-1 expression. For this purpose, we analyzed the expression of four SPI-1 genes (*invF*, *invH*, *sipB* and *sipC*) in isogenic *hilD* 3′UTR^+^ and *hilD* Δ3′UTR strains. Western blotting was used to monitor InvF, InvH, SipB and SipC levels using protein variants tagged with the 3xFLAG epitope. The levels of all four proteins were higher in *hilD* Δ3′UTR strains compared with the *hilD* 3′UTR^+^ background (Figure 4A), suggesting that the increased *hilD* mRNA level observed in the *hilD* Δ3′UTR strain yields a higher amount of HilD protein, which in turn results in increased SPI-1 expression. These observations were further confirmed by monitoring β-galactosidase activities of *invF::lac*, *invH::lac*, *sipB::lac* and *sipB::lac* fusions in *hilD* 3′UTR^+^ and *hilD* Δ3′UTR strains (Supplementary Figure S5). Hence, overproduction of *hilD* mRNA in the absence of its native 3′UTR correlates with SPI-1 overexpression, suggesting that the *hilD* 3′UTR may promote *hilD* mRNA turnover.

To define the region(s) of the *hilD* 3′UTR necessary for mRNA turnover, we constructed a set of mutants containing partial deletions of the 3′UTR. The mutants conserved 200, 150, 100, 50 and 25 nucleotides of the region immediately downstream of the *hilD* stop codon, followed by the 82 nt pKD13 scar and the Rho-independent transcriptional terminator (Supplementary Figure S6). We also constructed a mutant containing the 82-nt pKD13 scar immediately after the *hilD* stop codon, followed by the last (3′) 50 nucleotides of the 3′UTR and the transcriptional terminator (Supplementary Figure S6). SPI-1 expression was monitored by measuring the β-galactosidase activity of a *sipC::lac* fusion in the 3′UTR deletion mutants. Results shown in Figure 4B can be summarized as follows:

- The mutants containing 200, 150 and 100 nucleotides from the 5′ region of the 3′UTR showed *sipC* expression levels similar or identical to the wild type, indicating that deletion of downstream regions does not impair *hilD* mRNA turnover. Hence, signals for 3′UTR-mediated mRNA decay must be located within nucleotides 1–100.
- The mutants containing only 50 and 25 nucleotides of the 5′ region of the 3′UTR showed moderate increases in *sipC::lac* expression (2-fold and 3-fold, respectively), suggesting that some signals for *hilD* mRNA turnover had been lost upon deletion of nucleotides 25–50 and 50–100.
- In the mutant containing 25 nucleotides of the 3′UTR, *sipC::lac* expression was higher than in the wild type but lower than in the mutant lacking the whole 3′UTR (*hilD* Δ3′UTR) (Figure 4B), suggesting that signals for *hilD* mRNA turnover exist in the upstream (5′) 25 nucleotides of the 3′UTR.
- The *sipC::lac* expression level detected in the mutant containing the last 50 nucleotides of the 3′UTR (3′UTR_50end_) was similar to that of the whole-deletion mutant (*hilD* Δ3′UTR), supporting the view that downstream regions of the *hilD* 3′UTR do not play a relevant role in *hilD* mRNA turnover.
- A side, less relevant observation was that the length of the 3′UTR does not seem to be relevant for *hilD* mRNA turnover: mutants containing 50 nucleotides of the 3′UTR, either from the upstream region (3′UTR_50_) or the downstream region (3′UTR_50end_), have deletions of the same length but undergo disparate levels of *sipC* expression (Figure 4B and Supplementary Figure S6).
- The levels of *hilD* mRNA in the deletion mutants were analyzed by northern blotting (Supplementary Figure S7), and clear-cut correlations between mRNA abundance and SPI-1 expression levels were found.

In summary, conservation of the upstream (5′) 100 nucleotides of the 3′UTR is sufficient to keep *sipC::lac* expression at low levels, similar to those of the wild type, suggesting that signals for mRNA decay are contained within nucleotides 1–100 after the *hilD* stop codon.

### The *hilD* 3′UTR is a target for mRNA degradation by the RNA degradosome

**Figure 5.**
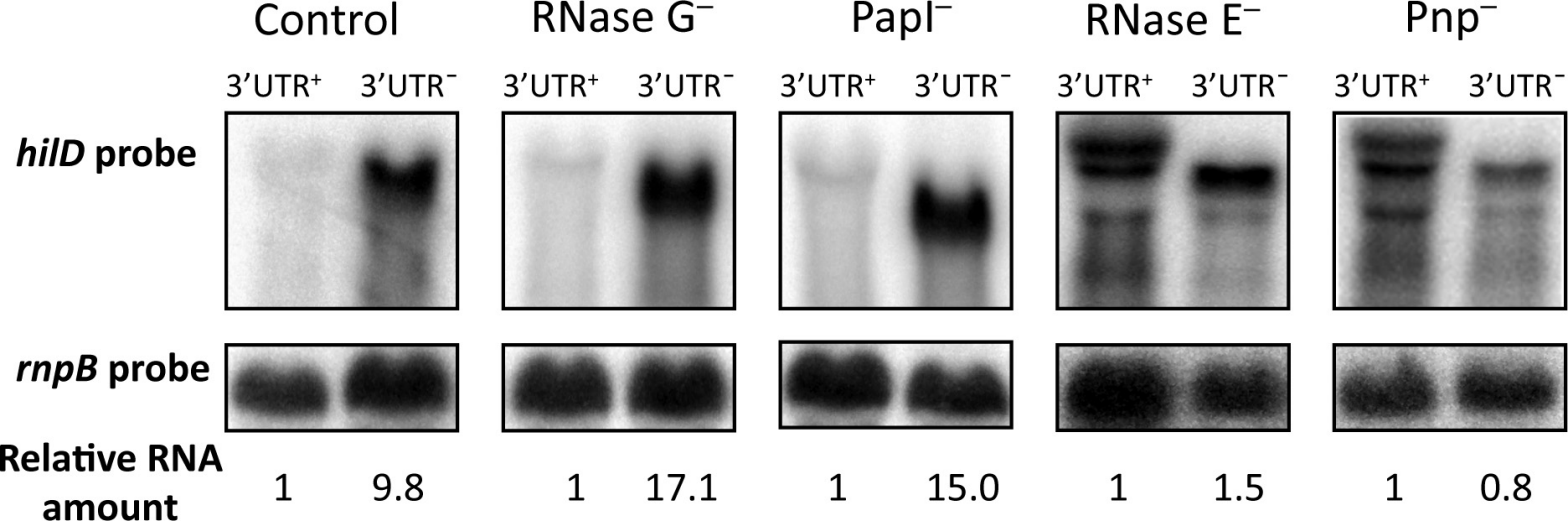
Northern blots of *hilD* and *hilD* Δ3′UTR mRNAs in *Salmonella enterica* strains lacking RNase G, PapI, RNase E, or Pnp. For simplicity, *hilD* and *hilD* Δ3′UTR mRNAs have been labeled 3′UTR^+^ and 3′UTR^−^, respectively. A riboprobe specific for *hilD* was used, and *rnpB* mRNA was used as an internal control. For quantification, the *hilD/rnpB* ratio was relativized to 1 in a 3′UTR^+^ background.

Because eukaryotic mRNA degradation often depends on long 3′UTRs (39,40), we hypothesized that the long 3′UTR of *hilD* mRNA might be a target for mRNA degradation by the RNA degradosome. If such was the case, the increased *hilD* mRNA level detected in the absence of the 3′UTR might be the consequence of reduced mRNA turnover. Hence, we reasoned, inactivation of factors involved in RNA degradation might suppress the differences observed in the level of *hilD* mRNA with and without its native 3′UTR. *Salmonella enterica* mutants carrying deletions in genes encoding ribonuclease E (RNase E), ribonuclease G (RNase G), polynucleotide phosphorylase (Pnp), or poly(A) polymerase I (PapI) were used. Because RNase E is essential in *S. enterica*, we used a mutant carrying a deletion that eliminates only the C-terminal region of the protein (41). The levels of *hilD* and *hilD Δ*3′UTR mRNAs were monitored in strains lacking RNase E, RNase G, Pnp or PapI. Increased levels of *hilD* Δ3′UTR mRNA were detected in the RNase G^−^ and PapI^−^ backgrounds (Figure 5), suggesting that the *hilD* 3′UTR is not involved in *hilD* mRNA degradation by RNase G or polyadenylation-dependent pathways. However, similar levels of *hilD* and *hilD* Δ3′UTR mRNAs were detected in strains lacking either RNase E or Pnp (Figure 5). These proteins are components of the RNA degradosome (42), suggesting that the *hilD* 3′UTR may be a target for mRNA degradation by the degradosome. Furthermore, the levels of *hilD* mRNA in mutants lacking RNase E and Pnp were higher than in the wild type (Figure 5).

*In silico* analysis of the *hilD* RNA sequence revealed six typical cleavage sites for RNase E [′-(A/G)N↓AU, where N identifies any nucleotide, and ↓ indicates the cleavage site (43)] in the upstream (5′) 100 nucleotides of the 3′UTR, with two putative sites contained in the upstream 25 nucleotides (Supplementary Figure S8). Altogether, our observations suggest that targeting of the upstream region of the *hilD* 3′UTR by the degradosome may contribute to maintain steady-state levels of *hilD* mRNA in the cell.

### The *hilD* mRNA level is modulated by Hfq in a 3′UTR-dependent manner

**Figure 6.**
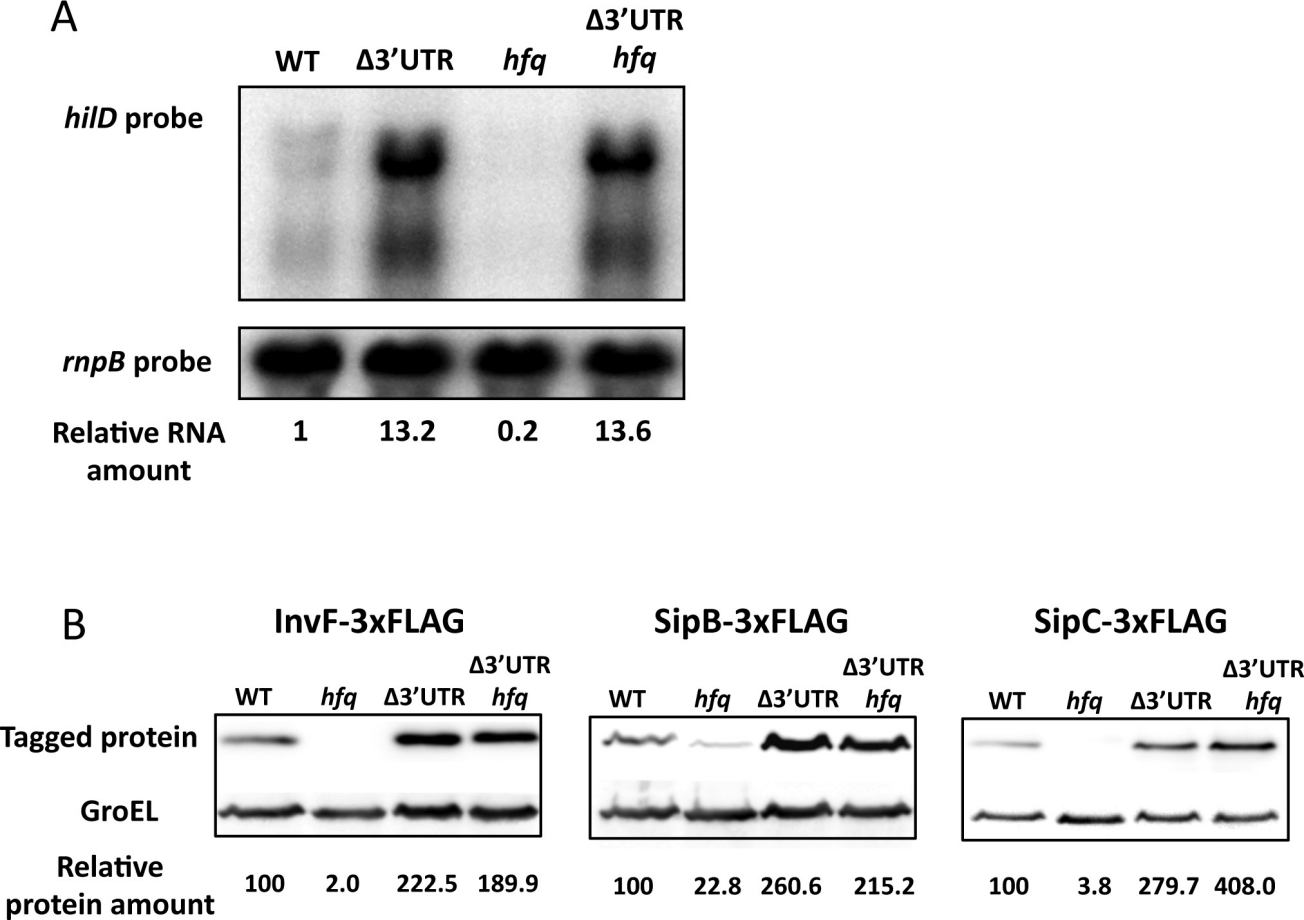
(**A**) *hilD* mRNA levels in Hfq^+^*hilD* 3′UTR^+^, Hfq^+^*hilD* 3′UTR^−^, Hfq^−^*hilD* 3′UTR^+^ and Hfq^−^*hilD* 3′UTR^−^ isogenic strains. *hilD* mRNA was detected by northern blot using a specific riboprobe, and *rnpB* mRNA was used as an internal control. For quantification, the ratio *hilD* mRNA/*rnpB* mRNA was relativized to 1 in Hfq^+^*hilD* 3′UTR^+^ background. (**B**) Western blots of InvF-3xFLAG, SipB-3xFLAG and SipC-3xFLAG in protein extracts from wild type, Δ3′UTR, Hfq^−^ and Δ3′UTR Hfq^−^ hosts. GroEL was used as loading control. For quantification, the ratio 3xFLAG-tagged protein/GroEL was relativized to 1 in the WT background.

A possibility suggested by the above results was that the 3′UTR of *hilD* mRNA might be a target for modulation of SPI-1 expression. On these grounds, we studied the role of the RNA chaperone Hfq. It has been reported that SPI-1 expression is repressed in mutants lacking Hfq (44). In addition, Hfq co-immunoprecipitation experiments have shown that Hfq binds *hilD* mRNA (45). To determine whether *hilD* expression was under Hfq control, we examined the levels of *hilD* mRNA in isogenic Hfq^+^ and Hfq^−^ strains by northern blotting (Figure 6A, lanes 1 and 3). Lack of Hfq reduced the level of *hilD* mRNA almost 5-fold. However, when the 3′UTR was deleted, similar levels of *hilD* Δ3′UTR mRNA were detected in Hfq^+^ and Hfq^−^ backgrounds (Figure 6A, lanes 2 and 4), indicating that the 3′UTR of *hilD* mRNA is necessary for modulation of *hilD* expression by Hfq. Next, we examined whether modulation of SPI-1 expression by Hfq depended on the *hilD* 3′UTR. For this purpose, we measured InvF, SipB and SipC protein levels in Hfq^+^ and Hfq^−^ backgrounds, either in the presence or in the absence of the *hilD* 3′UTR. As expected, protein levels were lower in Hfq^−^ hosts when the *hilD* 3′UTR was intact (Figure 6B). However, when the *hilD* 3′UTR was deleted, similar protein levels were detected in Hfq^+^ and Hfq^−^ backgrounds (Figure 6B), indicating that the 3′UTR of *hilD* mRNA is involved in modulation of SPI-1 expression by Hfq.

### The *hilD* 3′UTR is necessary to keep low levels of SPI-1 expression under non-invasive conditions

**Figure 7.**
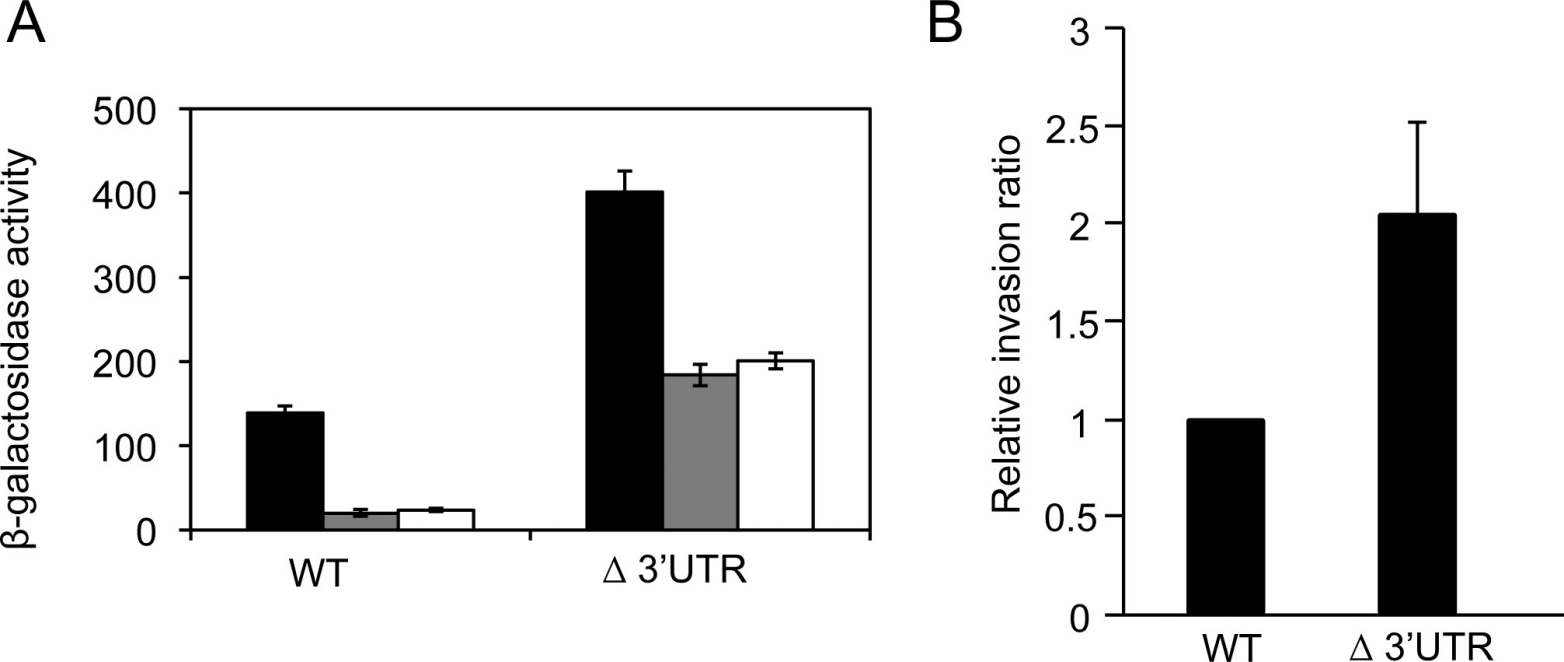
(**A**) β-Galactosidase activity of an *invF::lac* fusion in *hilD* 3′UTR^+^ and *hilD* Δ3′UTR hosts grown under different conditions: (i) standard conditions (black bars); (ii) exponentially growing cultures with high aeration (gray bars); and (iii) stationary phase cultures grown under low osmolarity (LB without salt). (**B**) Relative invasion ratios of epithelial HeLa cells by *Salmonella enterica* serovar Typhimurium carrying either the native *hilD* 3′UTR (wild-type strain ATCC 14018) or the *hilD* Δ3′UTR allele (*hilD* Δ3′UTR Km^r^, strain SV6189). The invasion ratio was determined 2 h post-infection and is expressed as the ratio between viable intracellular bacteria and viable bacteria added to the HeLa cell culture. For simplicity, ratios were normalized to the infection ratio of ATCC 14028. Each experiment was run in triplicate, and the data represented are averages of five independent experiments, with standard deviations shown as error bars. The difference in invasion ratios was significant (*P* < 0.05) according to Student's *t* test.

A standard practice in studies of *Salmonella* invasion and SPI-1 expression is growth in LB broth without aeration until stationary phase (22). These conditions intend a reductionist imitation of the animal intestine, and permit efficient activation of SPI-1 expression. Under ‘non-invasive’ conditions, SPI-1 expression is low or absent (8,9). On these grounds, we wondered whether degradosome targeting of the *hilD* 3′UTR might play a role in SPI-1 repression under non-invasive conditions. We monitored expression of an *invF::lac* fusion in *hilD* 3′UTR^+^ and *hilD* Δ3′UTR strains grown under two conditions in which SPI-1 is known to be partially or completely repressed: exponential growth with high aeration and growth at low osmolarity (LB without salt). Absence of the *hilD* 3′UTR caused a strong increase in *invF* expression under non-invasive conditions (Figure 7A), suggesting that the 3′UTR of *hilD* mRNA may contribute to keep SPI-1 repressed under conditions that are not appropriate for invasion.

To investigate whether expression of SPI-1 genes under non-invasive conditions permitted *Salmonella* invasion, we examined the effect of *hilD* 3′UTR deletion on the invasion rate of epithelial cells *in vitro*. Cultured HeLa cells were infected with wild-type *S. enterica* ATCC 14028 and with the *hilD* Δ3′UTR mutant grown in LB to exponential phase. The infection ratios of ATCC 14028 were extremely low (between 10^−4^ and 10^−6^), as expected under non-invasive conditions. However, the *hilD* Δ3′UTR mutant showed a higher invasion rate (Figure 7B).

## DISCUSSION

Certain eukaryotic mRNAs have long 3′UTRs (36) that modulate stability, translation and/or localization (39,46–48). In prokaryotes, the traditional notion is that 3′UTRs mainly harbor a transcriptional terminator that contributes to RNA stabilization, preventing degradation by exonucleases (49–51). However, recent advances in transcriptomic analysis have identified long 3′UTRs in some bacterial transcripts (52–54), raising the possibility that prokaryotic 3′UTRs might have physiological roles like their eukaryotic counterparts (55). For example, in *Bacillus subtilis*, nine different mRNAs harbor a conserved 3′UTR of around 220 nucleotides whose functional significance remains to be confirmed (53). Furthermore, 3′UTR-derived sRNAs potentially involved in posttranscriptional regulation in *trans* have been found in *S. enterica* (56) and *E. coli* (57). A recent study has shown that translation of a *Staphylococcus aureus* mRNA is controlled by interaction between the 5′UTR and the 3′UTR (58). In support of the emerging view that prokaryotic 3′UTRs may have functional relevance, we describe that *S. enterica hilD* mRNA contains a 3′UTR of 310 nucleotides (Figure 1), and provide evidence that the 3′UTR is a *cis*-acting element for modulation of mRNA stability.

Increased levels of *hilD* mRNA are found in the absence of the 3′UTR (Figure 2), and *hilD* mRNA increase is suppressed in the absence of either RNase E or Pnp (Figure 5), suggesting that the *hilD* 3′UTR is a target for *hilD* mRNA degradation by the RNA degradosome. This hypothesis is consistent with a previous report indicating that *Salmonella* mutants lacking functional RNase E undergo increased SPI-1 expression (11). Furthermore, degradation of certain bacterial RNAs has been shown to involve endonucleolytic cleavages downstream the coding sequence (59–62).

The ability of the *hilD* 3′UTR to serve as a degradosome target is supported by its nucleotide composition, which fits well in the preference of RNase E to degrade RNAs rich in A and U residues (42). The *hilD* 3′UTR contains 64% of A–U nucleotides, a percentage that increases up to 85% in the first (5′) 60 nucleotides. Signals for degradosome-mediated turnover appear to be located within the upstream 100 nucleotides of the *hilD* 3′UTR, and the upstream (5′) 25 nucleotides seem to be especially relevant (Figure 4B). These tentative conclusions are supported by the occurrence of six putative RNase E cleavage sites in the upstream 100 nucleotides of the *hilD* 3′UTR (Supplementary Figure S8).

Although the above observations suggest that the *hilD* 3′UTR might be a direct target for the RNA degradosome, we have not been able to detect differences in the half-lives of *hilD* mRNA with and without 3′UTR (data not shown), opening the possibility for alternative, more indirect models. One such model is the involvement of an unstable small RNA (sRNA) in the control of *hilD* mRNA turnover. Stopping transcription with rifampicin would cause fast disappearance of the sRNA, and the sRNA effect on mRNA stability would be masked. It seems also likely that the *hilD* mRNA increase detected in the absence of the 3′UTR (∼11-fold, Figure 2B) may have a transcriptional component due to autogenous control of *hilD* transcription and the positive feedback loop that governs SPI-1 expression (37,38). In agreement with this prediction, we observe a smaller increase (3.5-fold) when *hilD* is transcribed from a heterologous promoter (Figure 2C). Hence, it is conceivable that the 3′UTR may have a modest impact on the stability of *hilD* mRNA but its effect is amplified by a positive feedback loop at the transcriptional level (38).

This study provides evidence also that the *hilD* 3′UTR may play an additional role in SPI-1 expression by serving as target for the Hfq RNA chaperone. Deletion of the *hilD* 3′UTR suppresses regulation of *hilD* and SPI-1 by Hfq (Figure 6), suggesting that the *hilD* 3′UTR is necessary for SPI-1 regulation by Hfq. The involvement of Hfq in *hilD* regulation further strengthens the possibility that the *hilD* 3′UTR might be the target for a sRNA. A previous study in *E. coli* has proposed that the sRNA GadY can stabilize *gadX* mRNA by direct interaction with its 3′UTR (63). If a similar mechanism is involved in control of *hilD* mRNA stability, it would explain the reduced levels of *hilD* mRNA found in Hfq^−^ mutants and the suppression of this decrease when the *hilD* 3′UTR is deleted.

The relevance of the *hilD* 3′UTR in *Salmonella* physiology is illustrated by the phenotypic consequences of *hilD* 3′UTR deletion. Mutants lacking the *hilD* 3′UTR form small colonies on LB plates, and show retarded growth in liquid medium (Supplementary Figure S9). Growth retardation is a well known consequence of SPI-1 overexpression (64), probably because of the burden of building the TTSS machinery. Like other pathogenicity islands, SPI-1 is a gene cluster acquired by horizontal transfer whose accomodation into the host regulatory network requires the concurrence of multiple controls. Transcriptional regulators encoded in SPI-1 itself and in core regions of the chromosome relieve H-NS-mediated silencing and adjust transcription to appropriate environmental circumstances (8–10). HilD is one of the main transcriptional regulators of SPI-1. Although the *hilD* gene itself is subjected to transcriptional control, most regulatory systems that control SPI-1 expression through *hilD* appear to act at the posttranscriptional or the postranslational levels (10), and the underlying mechanisms are incompletely understood. Targeting of the *hilD* 3′UTR by the degradosome may contribute to maintain steady-state levels of *hilD* mRNA, thus contributing to cellular homeostasis. Efficient turnover of *hilD* mRNA may be especially relevant under non-invasive conditions, when the growth retardation caused by SPI-1 expression is not compensated by the possibility of colonizing an animal niche.

The abundance of RNase E targets in the upstream 100 nucleotides of the 3′UTR and the evidence that this region is sufficient for *hilD* RNA turnover may fuel the speculation that downstream regions of the 3′UTR might contain additional regulatory signals. For instance, Chao *et al.* have described that the 3′UTR region of *hilD* mRNA appears enriched upon Hfq co-inmunoprecipitation, defining a candidate region for a new 3′UTR-derived sRNA (56). Hence, the *hilD* 3′UTR might not only provide a target for *hilD* mRNA turnover but be also the source of a sRNA. In support of this speculation, certain 3′UTRs are known to be a source of small regulatory RNAs in *S. enterica* (56). This outlook, at this stage speculative, may further increase the interest of the *hilD* 3′UTR as a prokaryotic example of a 3′UTR with functional capacity.

## SUPPLEMENTARY DATA

Supplementary Data are available at NAR online.

SUPPLEMENTARY DATA
