# Supplementary material for: A eukaryotic-like 3′ untranslated region in Salmonella enterica hilD mRNA
Source: Nucleic Acids Res. 2014 Mar 20;42(9):5894–906. doi: 10.1093/nar/gku222 (PMC4027200; doi:10.1093/nar/gku222)
Supplement: SUPPLEMENTARY DATA [file supp_42_9_5894__index.html]

A eukaryotic-like 3′ untranslated region in Salmonella enterica hilD mRNA — A eukaryotic-like 3′ untranslated region in Salmonella enterica hilD mRNA — SUPPLEMENTARY DATA 

# A eukaryotic-like 3′ untranslated region in *Salmonella enterica hilD* mRNA

## SUPPLEMENTARY DATA

**Files in this Data Supplement:**

- SUPPLEMENTARY DATA
