# Supplementary material for: A eukaryotic-like 3′ untranslated region in Salmonella enterica hilD mRNA
Source: Nucleic Acids Res. 2014 Mar 20;42(9):5894–906. doi: 10.1093/nar/gku222 (PMC4027200; doi:10.1093/nar/gku222)
Supplement: SUPPLEMENTARY DATA [file supp_gku222_nar-01629-a-2013-File009.pdf]

A eukaryotic-like 3' untranslated region in *Salmonella enterica* *hilD* mRNA

Javier López-Garrido, Elena Puerta-Fernández, Josep Casadesús

SUPPLEMENTARY DATA

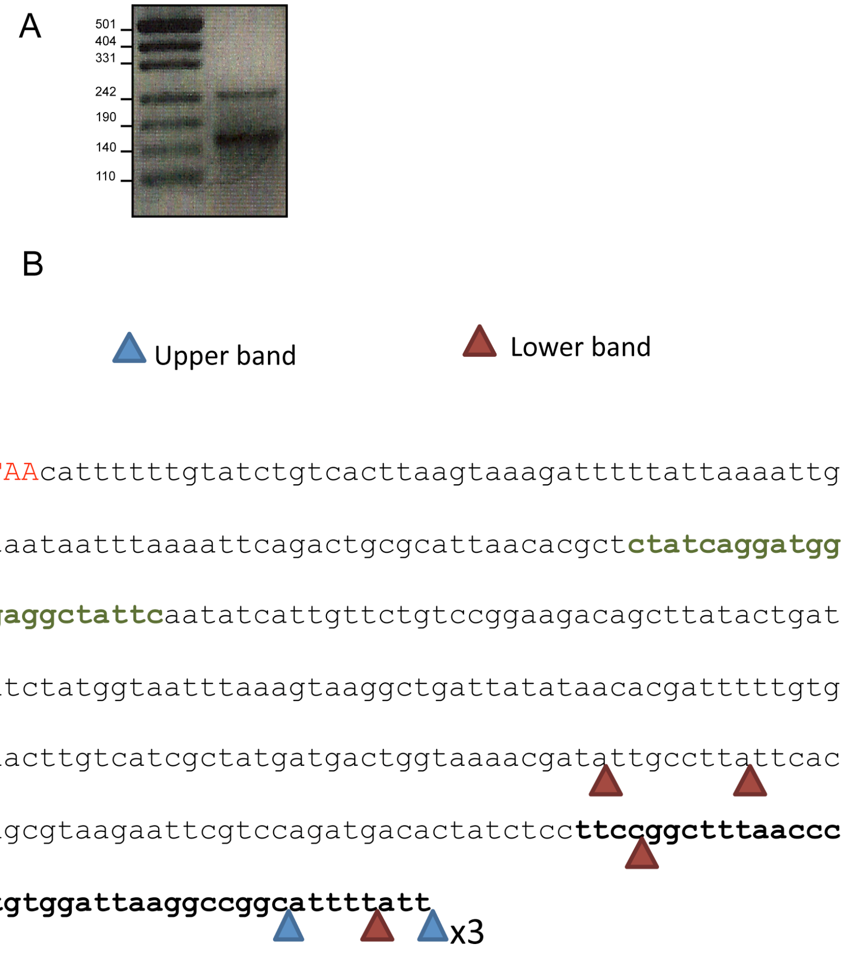

Figure S1. A. 3'RACE of *hilD* mRNA. Two different fragments appear after PCR amplification, identified by a sharp band slightly over 242 nt and a fainter band between 140 and 190 nt. Both fragments were purified and cloned on the pTOPO vector, and four independent clones were sequenced for each fragment. Their specific 3'ends of are shown in panel B. **B.** Blue arrowheads mark the 3' end of the four different clones of the higher band. Three such clones map at the same point, right after the U-rich region of the Rho-independent transcriptional terminator (sequence in bold), and one maps 8 nt upstream. Red arrowheads indicate the 3' end of the clones of the lower band. They all map in different positions along the 3'UTR, suggesting that they may be degradation intermediates. The *hilD* stop codon is shown in red capital letters, and the oligonucleotide used for 3'RACE is outlined in green.

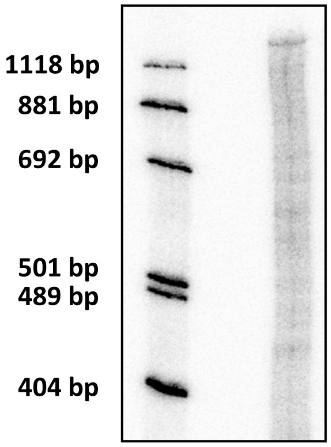

Figure S2. Northern blot of *hilD* mRNA in a 4.5% acrylamide gel. 10 µg of total RNA from *S. enterica* 14028 was loaded in a 4.5% polyacrylamide gel (right lane), transferred onto a Hybond-XL nitrocellulose membrane (GE Healthcare) and hybridized with a P^{32}-labelled riboprobe specific for the upstream (5') 300 nucleotides of the *hilD* coding sequence. A sharp band of a size higher than 1118 nucleotides is observed, together with a smear probably due to RNA degradation. 5'-end labelled DNA fragments obtained from pUC57 digestion with HindIII and pUC19 digestion with MspI and DraI were used as molecular weight size marker.

```
sv. Typhimurium    TAAcattttttgtatctgtcacttaagtaaagatttttattaaaa-ttgtaataatttaaaattcagactgcgc
sv. Enteritidis    TAAcattttttgtatctgtcacttaagtaaagatttttattaaaa-ttgtaataatttaaaattcagactgcgc
sv. Typhi          TAAca-tttttgtatcagtcactcaagtaaagatttttattaaaa-ttttaataatttaaaattcagaccgtgc
subsp. salamae     TAAcattttg-atacctattgctcaagaaaatacttttattaaaa---------atttaaaattctgaccattt
subsp. arizonae    TAAcattttt-gggtttatcactcaagaaaagattttcattaaaa-ttttagcaatttaaaattctgaacactt
subsp. houtenae    TAAcattttt-ggttgtatcactcaagaaaatatttttattaaaa-tcttaataatttaaaattctgaacactt
Salmonella bongori TAAcattttttcgtatttattactcaagaaaatatttttattaaaacttttttcatttaacattataaacgctt
                   *********         *  ** *** *** * *** *******         ****** ***   *

sv. Typhimurium    attaacacgctctatcaggatgggaggctattcaatatcattgttctgtccggaagacagcttatactgatatc
sv. Enteritidis    attaacacgctctatcaggatgggaggctattcaatatcattgttctgtccggaagacagcttatactgatatc
sv. Typhi          attaacacgctatatcaggatgggaggctattcaacatcattgttctgtccggaagacagcttatactgatatc
subsp. salamae     attaacgtgttatagcaggacaggaggctattcaacatcattgttctgcccagaagataggctgtactgatatc
subsp. arizonae    tataacacaccataataggattggaggttattcaacatcattgttctgtccagaagacagtccgtactgatatc
subsp. houtenae    cataaaacgccataataggactggaggttattcaatatcattgttctgtccggaagacagcctgcactgttatc
Salmonella bongori attaatacgctatagtaggatgggaggctatttaacattgttgttctgtccaaaagacagcctgcattgatatc
                     ***       **  ****  ***** **** ** **  ******** **  **** **     * ** ****

sv. Typhimurium    tatggtaatttaaagtaaggctgattatataacacgatttttgtgaacttgtcatcgctatgatgactggtaaa
sv. Enteritidis    tctggtaatttaaagtaaggctgattatataacacgatttttgtgaacttgtcatcgctatgatgactggtaaa
sv. Typhi          tctggtaatttaaagtaaggctgattatataacacgatttttgtgaacttgtcatcgctatgatgactggtaaa
subsp. salamae     tcttgcaatttaaagtaaggctgattatacaacacgatttttgtgaacttgtcatcgctatgatgacgggtaaa
subsp. arizonae    tctgacaatttaaagcaaggctgttcttacaacgcgacttttgtgaactcgtcatcgctatgatgactggtaaa
subsp. houtenae    tctggaaatttaaagcaaggctgattatacaacgcaatttttgtgaactcgtcatcgtcatgatgactggtaaa
Salmonella bongori tctggcaatttactgtaaggctgattatgcaacgcgatttttgtgaacttgtcatcgctatgatgacgggtaaa
                   * *   ******  * ******* *  *  *** * * *********** *******  ******** ******

sv. Typhimurium    acgatattgccttattcacatcgtaagaattcgtccagatgacactatctccttccggctttaaccctgtggat
sv. Enteritidis    acgatattgccttattcacagcgtaagaattcgtccagatgacactatctccttccggctttaaccctgtggat
sv. Typhi          ccgatattgccttattcacagcgtaagaatccgtccagatgacactatctccttccggctttaaccctatggat
subsp. salamae     ctgatattacctcattcacagcgtaagaatctgtccggatgaaactatctctttccggctttagccctatggac
subsp. arizonae    cctatattaccttattcacagcgtgaaaatctgtccagatgaaagtatctccttccggctttaaccctgtggac
subsp. houtenae    ccaatattaccttattcacagcgtgagaatctgtccagatgaaactatctccttccggctttaaccctgtggtc
Salmonella bongori ccaatattaccttattcacagcgtgagaatctgtccagatgaaactatcttcttccggctttagccctgtggac
                      ***** *** ******* *** * ***  **** ***** * *****  *********** **** ***

sv. Typhimurium    taaggccggcattttatt
sv. Enteritidis    taaggccggcattttatt
sv. Typhi          taaggccggcattttatt
subsp. salamae     taaagccggcattttatt
subsp. arizonae    taaagccggcattttatt
subsp. houtenae    taaagccggcattttatt
Salmonella bongori taa-gccggcattttatt
                   *** **************
```

Figure S3. Alignment of the 3'UTR region of *Salmonella enterica* and *Salmonella bongori* serovars. An asterisk indicates nucleotides that are conserved in all sequences. Bold letters indicate the sequence of the Rho-independent transcriptional terminator.

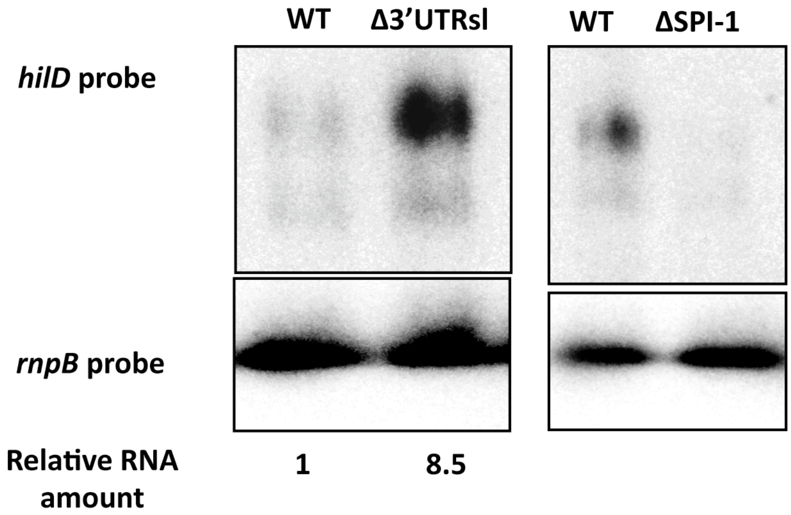

Figure S4. Northern blots of *S. enterica* ATCC 14028, *hilD* Δ3'UTRsl (SV8082) and ΔSPI-1 (SV6019) strains. A *hilD* riboprobe was used to detect *hilD* mRNA. *rnpB* mRNA was used as loading control. For quantification, the ratio *hilD/rnpB* was normalized to 1 in *hilD* mRNA carrying a native 3'UTR.

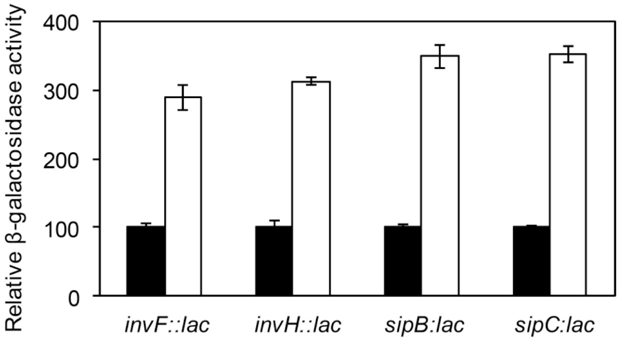

Figure S5. β-galactosidase activities of *invF::lac*, *invH::lac*, *sipB::lac*, and *sipC::lac* fusions in strains with a native *hilD* 3'UTR (black histograms) or carrying the *hilD* Δ3'UTR allele (white histograms). Due to the disparate activities of the fusions, β-galactosidase activities have been normalized to 100 in the 3'UTR$^+$ background.

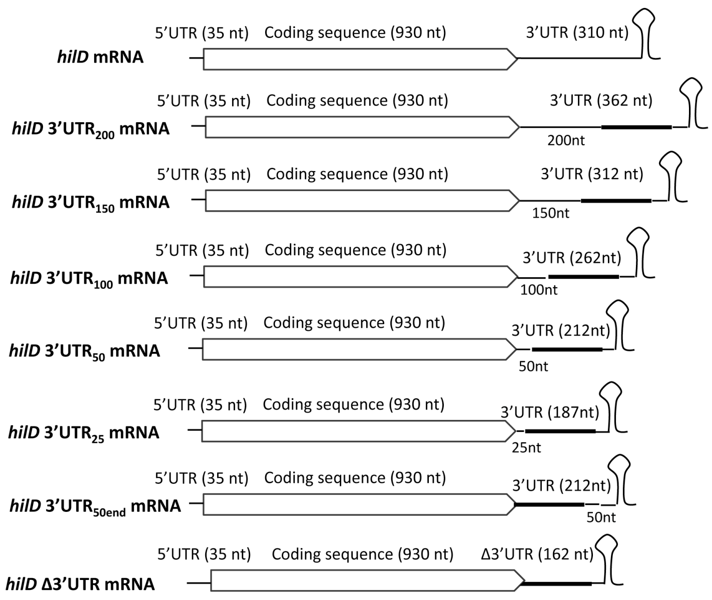

Figure S6. Diagrams of the primary structure of *hilD* mRNA in the 3'UTR deletion mutants constructed for this study. The thick line in the 3'region of *hilD* mRNA represents the 82-nt pKD13 scar. The Rho-independent transcriptional terminator, which is is left intact in all constructs, is also shown.

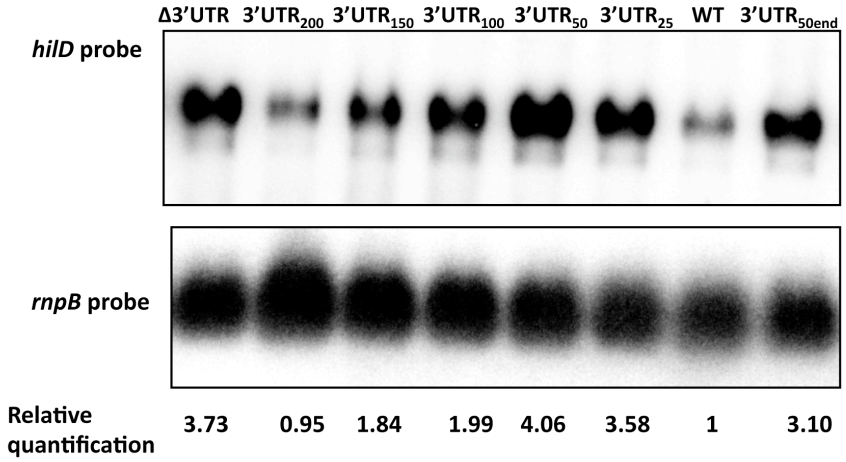

Figure S7. Northern blots of *hilD* mRNA in isogenic strains carrying mutations in the *hilD* 3'UTR region. For quantification, the ratio *hilD/rnpB* was normalized to 1 in *hilD* mRNA carrying a native 3'UTR (WT).

UAA<u>cauuuuuu**guau**cugucacuuaa**gua**aagauuuuuau</u>

<u>ua**aaau**uguaauaauuua**aaau**ucagacugc**gcau**uaaca</u>

<u>cgcucuauca**ggau**gggaggcua</u>uucaauaucauuguucu

guccggaagacagcuuauacugauaucuagguaauuuaa

aguaaggcugauuauauaacacgauuuuugugaacuuguc

aucgcuaugaugacugguaaaacgauauugccuuauucac

agcguaagaauucguccagaugacacuaucuccuu**ccggc**

uuuaacccuguggauuaaggccggcauuuuauu

Figure S8. Nucleotide sequence of the *hilD* 3'UTR indicating putative RNase E cleavage sites (A/GN↓AU, shown in red) for the first 100 nucleotides of the 3'UTR (underlined). Bold, uppercase UAA represents the *hilD* stop codon, and the sequence in bold at the end indicates the transcriptional terminator shown in Fig. 1. The second putative RNase E cleavage site depicted (GUA) can be expected to yield a putative cleavage site (GUAU) in the mutant containing 25 nucleotides of 3'UTR, after the pKD13 scar has been generated.

A

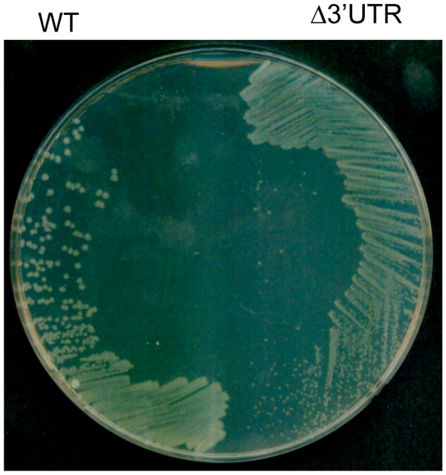

B

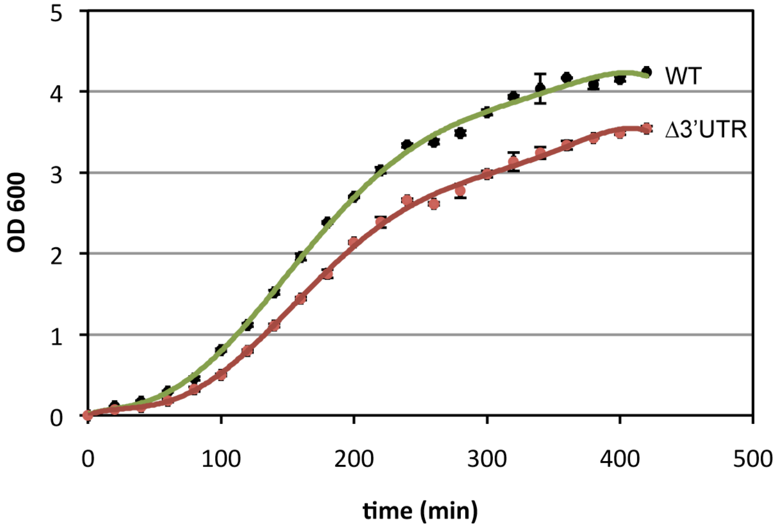

Figure S9. **A.** Colonies of ATCC 14028 (left) and the *hilD*Δ3'UTR strain SV6190 (right) on an LB agar plate. **B**. Growth curves of ATCC 14028 and SV6190 (*hilD*Δ3'UTR) in LB broth at 37 ºC with shaking at 200 rpm (averages and standard deviations from three independent experiments).

Table S1. Strain list

Strain designation	Genotype or description	Reference or source
ATCC 14028	Wild type	American Type Culture Collection
SV5293	Φ(*sipC'-lacZ*$^+$)	(Lopez-Garrido and Casadesus 2010)[a]
SV5297	Φ(*invF'-lacZ*$^+$)	(Lopez-Garrido and Casadesus 2010)[a]
SV5301	Φ(*invH'-lacZ*$^+$)	(Lopez-Garrido and Casadesus 2010)[a]
SV5382	Φ(*sipB'-lacZ*$^+$)	(Lopez-Garrido and Casadesus 2010)[a]
SV5457	*invF*::3xFLAG	(Lopez-Garrido and Casadesus 2010)[a]
SV5458	*invH*::3xFLAG	This study
SV5459	*sipB*::3xFLAG	This study
SV5460	*sipC*::3xFLAG	This study
SV5646	*hfq::Km*^{r}	This study
SV5961	Δ*rne*::*cat*	(Lopez-Garrido and Casadesus 2010)[a]
SV5963	Δ*pnp*::*cat*	(Lopez-Garrido and Casadesus 2010)[a]
SV6019	Δ*SPI-1* (Km^r)	F. Baisón-Olmo
SV6189	*hilD* Δ3'UTR (Km^r)	This study
SV6190	*hilD* Δ3'UTR	This study
SV6192	*hilD* Δ3'UTR *hfq::Km*^{r}	This study
SV6227	*hilD* Δ3'UTR Φ(*invF'-lacZ*$^+$)	This study
SV6636	P_{LtetO}-*hilD*	This study
SV6637	P_{LtetO}-*hilD* Δ3'UTR	This study
SV6639	*hilD* Δ3'UTR Δ*pnp*::*cat*	This study
SV6640	*hilD* Δ3'UTR Δ*rne*::*cat*	This study
SV6642	*hilD* Δ3'UTR *invF*::3xFLAG	This study

SV6643	*hfq::Cm*^{r} *invF*::3xFLAG	This study
SV6644	*hilD* Δ3'UTR *hfq::Cm*^{r} *invF*::3xFLAG	This study
SV6645	*hilD* Δ3'UTR *invH*::3xFLAG	This study
SV6646	*hilD* Δ3'UTR Φ(*sipB'-lacZ*$^+$)	This study
SV6648	*hilD* Δ3'UTR *sipB*::3xFLAG	This study
SV6649	*hfq::Cm*^{r} *sipB*::3xFLAG	This study
SV6650	*hilD* Δ3'UTR *hfq::Cm*^{r} *sipB*::3xFLAG	This study
SV6651	*hilD* Δ3'UTR Φ(*sipC'-lacZ*$^+$)	This study
SV6653	*hilD* Δ3'UTR *sipC*::3xFLAG	This study
SV6654	*hfq::Cm*^{r} *sipC*::3xFLAG	This study
SV6655	*hilD* Δ3'UTR *hfq::Cm*^{r} *sipC*::3xFLAG	This study
SV6656	*hilD* Δ3'UTR Φ(*invH'-lacZ*$^+$)	This study
SV6789	Δ*rng*::*cat*	This study
SV6790	*hilD* Δ3'UTR Δ*rng*::*cat*	This study
SV6791	Δ*pcnB*::*cat*	This study
SV6792	*hilD* Δ3'UTR Δ*pcnB*::*cat*	This study
SV7440	14028/pIZ1988 (GFP-3'UTR)	This study
SV7441	14028/pIZ1989 (GFPΔ3'UTR)	This study
SV7600	*hilD*3'UTR$_{200}$	This study
SV7601	*hilD* 3'UTR$_{150}$	This study
SV7602	*hilD* 3'UTR$_{100}$	This study
SV7603	*hilD* 3'UTR$_{50}$	This study
SV7604	*hilD* 3'UTR$_{25}$	This study
SV7896	*hilD* 3'UTR$_{50end}$	This study
SV8082	*hilD*Δ3'UTRsl	This study

[a] J. López-Garrido & J. Casadesús, *Genetics* **184**: 637-649 (2010)

Table S2. Oligonucleotides used in this study (5'→3')

Oligonucleotide	Sequence
hilDriboprobeUP	atggaaaatgtaacctttgtaag
hilDriboprobeDO	gtttttttaatacgactcactatagggaggtatatcgaaatccatgtggc
rnpBriboprobeUP	gaagaagtgaaactgaccgataagc
rnpBriboprobeDO	taatacgactcactataggccgaagctgaccagacagtcg
JVO5527	ctatcaggatgggaggctattc
hilD-E2’	atcatcctcaggctggctcc
RT-hilD3’-UP	agcttacggatgttgccgatc
JVO5462	aactacgccatcgacattcataaaaatggcgaaccattaaattccggggatccgtcgacc
JVO5463	ggagatagtgtcatctggacgaattcttacgctgtgaatagtgtaggctggagctgcttc
rngUP	accgaattcgcgtcgactcacgtctgacctatgagtcgctcatatgaatatcctccttag
rngDO	cggctgatggccactgtaatgctccagcttactggtcatttgtaggctggagctgcttcg
rng-E1	ggtgcatacggtattcaggg
rng-E2	tcgtcgagattacggtgtcc
pcnBUP	gcccgtctgccgtaatgcgaaagacatagagtaagttgatcatatgaatatcctccttag
pcnBDO	tgggatgttccatcagtttccaggcgcgtttgccctgacgtgtaggctggagctgcttcg
pcnB-E1	cgctgagctatgattagccg
pcnB-E2	tccggtctaatgacgcaagc
hilD200UP	aaggctgattatataacacgatttttgtgaacttgtcatcattccggggatccgtcgacc
hilD150UP	ttgttctgtccggaagacagcttatactgatatctatggtattccggggatccgtcgacc
hilD100UP	cagactgcgcattaacacgctctatcaggatgggaggctaattccggggatccgtcgacc
hilD50UP	atctgtcacttaagtaaagatttttattaaaattgtaataattccggggatccgtcgacc
hilD25UP	atggcgaaccattaacattttttgtatctgtcacttaagtattccggggatccgtcgacc
hilD50endDO	gttttaccagtcatcatagcgatgacaagttcacaaaaatgtgtaggctggagctgcttc
EPF043	gcggctagcaaaggagaagaacttttcactgg

EPF044	cagatacaaaaaatgttatttgtagagctcatcc
EPF045	gagctctacaaataacatttttttgtatctgtcacttaag
EPF046	gcggctagctgcctggcagaaagctaacaagc
EPF047	gaccatccccggaatttatttgtagagctcatcc
EPF048	gagctctacaaataaattccggggatccgtcgacc
EPF098	cttacgatgtgaatatagcctcccatcctgatagagcg
EPF103	cttacgatgtgaatattaatggttcgccatttttatg
EPF104	atggcgaaccattaatattcacatcgtaagaattcg
